# Strength Investigation and Prediction of Superfine Tailings Cemented Paste Backfill Based on Experiments and Intelligent Methods

**DOI:** 10.3390/ma16113995

**Published:** 2023-05-26

**Authors:** Yafei Hu, Keqing Li, Bo Zhang, Bin Han

**Affiliations:** 1 School of Civil and Resource Engineering, University of Science and Technology Beijing, Beijing 100083, China; b20190027@xs.ustb.edu.cn (Y.H.); likeqing@ustb.edu.cn (K.L.); s20200012@xs.ustb.edu.cn (B.Z.); 2 Key Laboratory of Ministry of Education of China for Efficient Mining and Safety of Metal Mines, University of Science and Technology Beijing, Beijing 100083, China

**Keywords:** superfine tailings, filling mining, underflow productivity, machine learning, strength prediction

## Abstract

The utilization of solid waste for filling mining presents substantial economic and environmental advantages, making it the primary focus of current filling mining technology development. To enhance the mechanical properties of superfine tailings cemented paste backfill (SCPB), this study conducted response surface methodology experiments to investigate the impact of various factors on the strength of SCPB, including the composite cementitious material, consisting of cement and slag powder, and the tailings’ grain size. Additionally, various microanalysis techniques were used to investigate the microstructure of SCPB and the development mechanisms of its hydration products. Furthermore, machine learning was utilized to predict the strength of SCPB under multi-factor effects. The findings reveal that the combined effect of slag powder dosage and slurry mass fraction has the most significant influence on strength, while the coupling effect of slurry mass fraction and underflow productivity has the lowest impact on strength. Moreover, SCPB with 20% slag powder has the highest amount of hydration products and the most complete structure. When compared to other commonly used prediction models, the long-short term memory neural network (LSTM) constructed in this study had the highest prediction accuracy for SCPB strength under multi-factor conditions, with root mean square error (RMSE), correlation coefficient (R), and variance account for (VAF) reaching 0.1396, 0.9131, and 81.8747, respectively. By optimizing the LSTM using the sparrow search algorithm (SSA), the RMSE, R, and VAF improved by 88.6%, 9.4%, and 21.9%, respectively. The research results can provide guidance for the efficient filling of superfine tailings.

## 1. Introduction

Tailings are one of the largest contributors to bulk solid waste, yet they have a notably low comprehensive utilization rate [1,2,3]. Taking China as an example, its tailings production in 2021 was approximately 1.3 billion metric tons, with a utilization rate of less than 40%. Traditionally, tailings are deposited on the ground, and the heavy metals they contain are carried to the soil and groundwater by rain and wind, which causes serious damage to the ecological environment [4,5]. As countries around the world pay attention to ecological protection, the construction of green mines has become a major development trend in the mining industry. To reduce the environmental damage caused by tailings, scholars have successfully developed tailings cement filling technology, which significantly reduces stockpiles of ground tailings by filling the tailings into the underground mining area [6,7,8,9,10].

Although there have been multiple studies on tailings cemented filling technology, superfine-tailings cemented paste backfill (SCPB) still has problems including low utilization, low filling efficiency, high filling cost, and unstable filling quality [11,12]. To optimize the performance of SCPB, a large amount of research work has been carried out by scholars. Mineral admixtures such as slag powder, fly ash, and silicon powder have been used in SCPB with excellent results. These mineral admixtures have two main uses in SCPB: one is to optimize the grain size grade of the aggregates to improve the fluidity of the slurry; the other is to replace part of the cement with a supplementary cementitious material to improve the strength of filling body. Fly ash plays a key role in improving the fluidity of the slurry due to its fineness, ball effect, and lower requirement for water [13,14,15]. Slag powder is a potential supplementary cementitious material that promotes the formation of hydration products to improve the denseness and strength of SCPB in the later stages of the hydration process [16,17]. Silicon powder is a highly reactive volcanic ash material that has a great ability to absorb alkaline ions from the hydration products of SCPB [18]. In addition, the silicon powder has a filling effect, which prevents the slurry from bleeding, thereby improving the transport performance of SCPB slurry. The above studies show that mineral admixtures have the ability to optimize the performance of SCPB. In addition to mineral admixtures, the grain size grade of superfine-tailings also has a significant effect on SCPB performance, but there have been relatively few studies on the coupling effect of grain size grade of superfine-tailings and mineral admixtures.

The development of artificial intelligence has shown potential for application in engineering, including filling mining [19,20,21,22]. For example, machine learning is increasingly being applied for the prediction and optimization of the strength of filling body. Researchers have carried out related studies on the prediction of uniaxial compressive strength (UCS) of filling body, using artificial neural networks (ANN), support vector machines (SVM), and random forests [23,24,25,26]. High prediction accuracy was achieved by training, verifying, and combining experiments on different models [27,28,29,30,31,32,33]. In addition, optimization algorithms including a genetic algorithm (GA), particle swarm optimization (PSO), and gray wolf optimization (GWO) were used to optimize the hyperparameters of the above models, which significantly improved the predictive performance [27,28,29,30,31,32,33]. Machine learning has greatly improved the efficiency of filling body research. Compared with traditional laboratory experiments, it can save a large amount of manpower and resources.

In this study, response surface methodology (RSM) was applied to optimize the experimental design to investigate the mechanism and effect of slag powder dosage, slurry mass fraction, and underflow productivity (characterizing the grain size of tailings) on the UCS of SCPB. At the same time, microstructure and hydration product characteristics of SCPB were analyzed with the assistance of various microscopic testing methods. Then, the UCS prediction model of SCPB under multiple factors was constructed using long-short term memory neural network (LSTM). Finally, the LSTM was optimized based on various optimization algorithms to achieve high accuracy prediction of the UCS of SCPB.

The objective of this study is to investigate experimentally the impact patterns and mechanisms of different factors on superfine SCPB, and to achieve high-precision prediction of SCPB strength under the influence of multiple factors using intelligent algorithms. The results of the research have important implications for improving the utilization and filling effectiveness of superfine tailings and for developing efficient applications of machine learning in filling mining.

## 2. Materials and Methods

### 2.1. Materials

#### 2.1.1. Tailings

**Figure 1:**
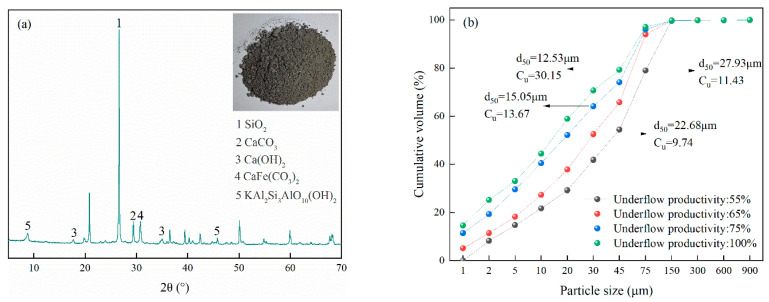
XRD results and particle sizes of tailings: (**a**) XRD results, (**b**) particle sizes.

In this research, tailings from a gold mine in Guizhou Province, China, were used as filling aggregates. The density and bulk density of the tailings were 2.65 g/cm^3^ and 1.58 g/cm^3^, respectively. The main chemical composition of the tailings is shown in Table 1 and Figure 1a. The activity index of the tailings, Ma = 0.04 < 1.00, indicates that it is basically inactive and cannot react chemically in an alkaline cementation system. From Figure 1b, it can be seen that the median particle size (d_50_) of the unclassified tailings (underflow productivity is 100%) from this gold mine is 12.53 μm, and particles less than 75 μm account for more than 90%, which indicates that the tailings are superfine and the UCS of the SCPB is low.

The gradation of the tailings is an important factor in determining the mix proportion of the SCPB, which has a significant effect on the UCS of the SCPB. To investigate the effect of tailings with different grain sizes on the UCS of SCPB, graded tailings with underflow productivity of 55%, 65%, and 75%, respectively, were prepared by cyclones at Haiwang company (Weihai, China). From Figure 1b, it can be seen that the median particle sizes of the 3 tailings were 27.93 μm, 22.68 μm, and 15.05 μm, and uniformity coefficients were 11.43, 9.74, and 13.67, respectively. In addition, the smaller the underflow productivity, the coarser was the particle size of the corresponding tailings. The underflow productivity is calculated by measuring the inflow mass fraction, the overflow mass fraction and the underflow mass fraction of the cyclone. The calculation equation is as follows:

(1) $$P=\frac{c_{d}(c_{i}-c_{0})}{c_{i}(c_{d}-c_{0})}\times 100\%$$

where *P* is the underflow productivity (%); *c_i_* is the inlet mass fraction (%); *c*_0_ is the overflow mass fraction (%); and *c_d_* is the underflow mass fraction (%).

#### 2.1.2. Composite Cementitious Materials

In this research, the cement and slag powder were selected to form a composite cementitious system. The parameters and properties of the materials are listed below.

**Figure 2:**
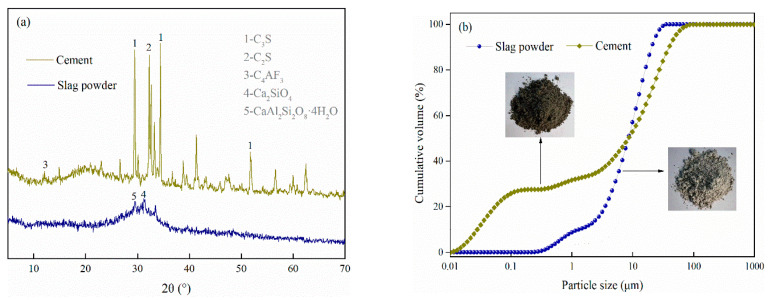
XRD results and particle sizes of cementitious materials: (**a**) XRD results, (**b**) particle sizes.

(1) This study employed cement as the binder, sourced from the same mine as the tailings to ensure experimental materials are in line with field conditions, thereby enhancing the applicability of the research. According to Figure 2b, it can be seen that the particle size of the cement is slightly smaller than that of the tailings, resulting in a larger specific surface area. This cement displays an initial setting time of 150 min, followed by a final setting time of 220 min. The 28 d compressive strength and flexural strength of the cement are 54.3 MPa and 8.7 MPa. As shown in Figure 2a, the main phase components of cement are C_3_S, C_2_S, and C_4_AF, indicating that the cement possesses strong hydrolytic capabilities.
(2) Slag powder was used as the supplementary cementitious and active material. It has a specific surface area of 440 m^2^/kg and a density of 2.9 g/cm^3^. As shown in Table 1, the alkalinity coefficient of slag powder M_0_ = 1.17 > 1; the quality coefficient K = 2.27 > 1.8; the activity coefficient M_a_ = 1.57 > 1 [34]. This indicates that it is an alkaline slag with high activity. From the XRD results in Figure 2, it is clear that the slag powder contains a few crystalline phase substances with low crystallinity, mainly calcium aluminosilicate, dicalcium aluminosilicate, dicalcium silicate, and calciclite feldspar.

### 2.2. Methods

**Figure 3:**
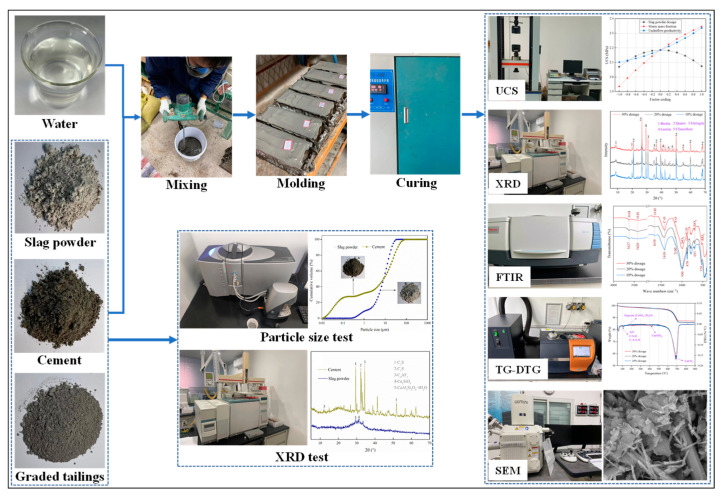
Experimental flow chart.

Several testing methodologies were employed to study the mechanical properties and formation mechanisms of SCPB. The testing procedures are illustrated in Figure 3.

(1) Uniaxial compressive strength test (UCS test): When the specimen was cured for a specific time, the UCS was tested using a WDW-2000 automatic pressure testing machine. To ensure the accuracy of the experimental results, three samples were prepared for each group of tests, and their UCS values were measured separately. The average value of the three samples was taken as the UCS of the corresponding mix proportion for that group.
(2) X-ray diffraction test (XRD test): SCPB specimens were ground into powder and analyzed for their mineral phases using an X-ray diffractometer to determine the fractions of hydration products under different affecting factors. The equipment model used for XRD was a Bruker D8 Focus Bragg-Brentano diffractometer. The XRD test was conducted within the 2θ range of 5~70°, with a data collection rate of 7 s/step and a step size of 0.03°.
(3) Fourier transform infrared spectroscopy test (FT-IR test): When a beam of infrared rays of different wavelengths is irradiated onto the molecules of a substance, an infrared absorption spectrum is formed because different substances absorb infrared rays of specific wavelengths [35,36]. Based on the spectral bands, the structural composition of the substance to be measured is inferred and the intensity of the absorption bands is used to obtain the content of the substance. The FT-IR testing was performed using a Bruker Tensor 27 instrument.
(4) Thermogravimetry and differential thermogravimetry test (TG-DTG test): The TG curve reflects the mass change of a substance with temperature, and the DTG curve represents the rate of change of the mass of a substance as a function of time and temperature [37,38]. When the mass loss is low, the steps on the TG curve are not significant. The TG-DTG testing was conducted using the STA409C high-temperature thermal analyzer developed by NETZSCH, a German company. At this time, the analysis needs to be performed using the DTG curve. In this test, the initial temperature was 20 °C and the temperature increase rate was 10 °C/min, stopping when it reached 900 °C.
(5) Scanning electron microscopy test (SEM test): SEM is a widely used method for observing microscopic structures. In this study, this method was employed to observe the distribution of pores in the microstructure of SCPB under different mix proportions. Additionally, Image J software was utilized to conduct grayscale analysis of the microstructure, in order to compare the compactness of different SCPB samples. Furthermore, SEM was utilized to observe the hydration products in SCPB, which aided in analyzing the degree of hydration reactions in different samples [39,40].

### 2.3. Experiment Design

The relationship between the mix proportion and the UCS of SCPB is highly complex and uncertain, exhibiting non-linear behavior. To investigate the impact of various factors on the UCS of SCPB, this study employed response surface methodology (RSM) for experimental design [41,42].

According to previous extensive exploratory experiments, the UCS of SCPB can be achieved according to the requirements of the construction site when the cement dosage is 15% of the solid material. The RSM experiments take the slag powder dosage (mass proportion of slag powder to cement), slurry mass fraction, and underflow productivity as the independent variables (denoted by *X*_1_, *X*_2_, and *X*_3_, respectively), and the UCS of SCPB at 7d, 14d, and 28d as the response values (denoted by *Y*_1_, *Y*_2_, and *Y*_3_, respectively). The experimental scheme and results are shown in Table 2 and Table 3, respectively.

## 3. Analysis of Experimental Results

### 3.1. UCS

#### 3.1.1. Single Factor Effect on UCS

**Figure 4:**
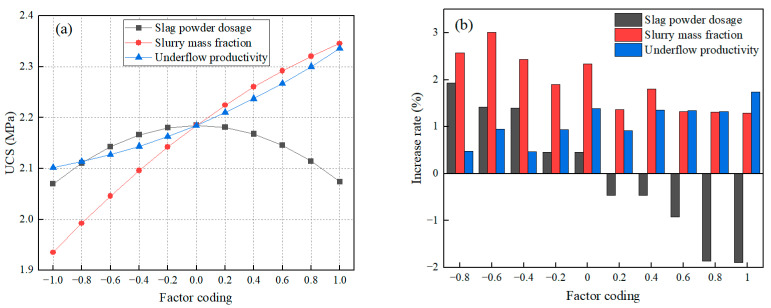
The effects of different single factors on UCS: (**a**) UCS, (**b**) increase rate.

The effects of different single factors on UCS are shown in Figure 4. With the increase of slag powder dosage, the UCS was first enhanced and then weakened. The increase rate of UCS became negative after the coded value of slag powder dosage exceeded 0 (i.e., slag powder dosage was more than 15%), indicating that the coded value of slag powder dosage higher than 0 negatively affected UCS. The enhancement of UCS increased with increasing slurry mass fraction but the increase rate gradually decreased, indicating that the enhancement trend of UCS gradually decreased with increasing slurry mass fraction. The enhancement of UCS increased with decreasing underflow productivity and the increase rate gradually increased, indicating that the enhancement trend of UCS gradually increased with decreasing underflow productivity. Overall, the coded value of 0 represented the turning point of the effect of each factor on UCS. When the coded value was higher than 0, the slag powder dosage had a negative effect on UCS, the slurry mass fraction had a weaker effect on the enhancement of UCS, and the underflow productivity had an enhanced effect on UCS.

#### 3.1.2. The Effect of Multifactor Coupling on UCS

**Figure 5:**
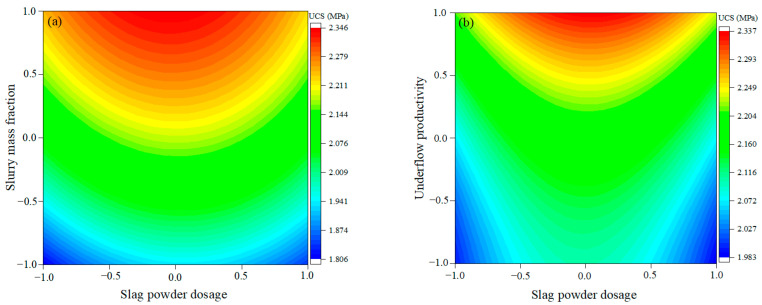

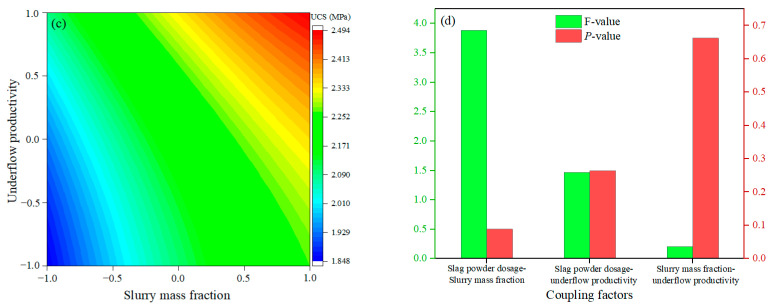
The effect of multifactor coupling on UCS: (**a**) slag powder dosage and slurry mass fraction, (**b**) slag powder dosage and underflow productivity, (**c**) underflow productivity and slurry mass fraction, (**d**) significance analysis.

The effect law of multifactor coupling on UCS is shown in Figure 5. From Figure 5a, it can be seen that the UCS followed a “concave” growth law under the coupling effect of slag powder dosage and slurry mass fraction. The centerline of the “concave” shape corresponds to a fly ash dosage of 20%. The UCS on this centerline is at its maximum in the horizontal direction. At the same time, the UCS increased gradually in the vertical direction with the increase of slurry mass fraction. The UCS corresponding to the different fitting proportions is symmetrically distributed along the centerline of the “concave” and gradually decreases from the centerline to the sides. From Figure 5b, it can be seen that UCS was enhanced and then weakened in the horizontal direction with the increase of slag powder dosage, and enhanced in the vertical direction with the decrease of underflow productivity. When the slag powder dosage was less than 15% or more than 25%, UCS increased slowly with the decrease of underflow productivity. When the slag powder dosage ranged from 15% to 25%, the UCS was enhanced significantly with the decrease of underflow productivity. From Figure 5c, it can be seen that the UCS was enhanced with the decrease of underflow productivity and the increase of slurry mass fraction. The UCS was enhanced by 13.1% with the slurry mass fraction at 65% and underflow productivity decreased from 75% to 55%. When the slurry mass fraction was at 71% and the underflow productivity decreased from 75% to 55%, the UCS was enhanced by 19.3%. This indicates that under the coupling effect of slurry mass fraction and underflow productivity, the larger the slurry mass fraction, the more significant the enhancement of underflow productivity on UCS. Figure 5d shows the significance analysis of the coupling effect of different factors on UCS. The higher the F-value and the smaller the *p*-value, the more significant the effect. From Figure 5d, it can be seen that the coupling effect of slag powder dosage and slurry mass fraction had the most significant effect on UCS, and the coupling effect of slurry mass fraction and underflow productivity has the lowest significance for UCS.

### 3.2. Micro Products

#### 3.2.1. XRD

**Figure 6:**
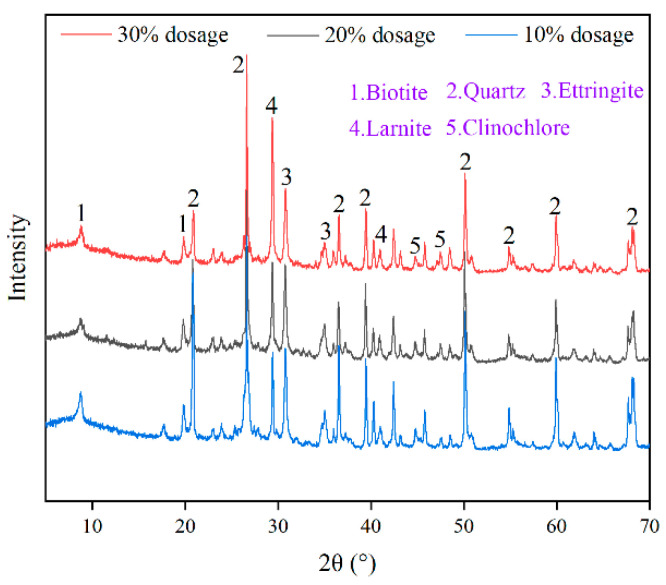
XRD results of SCPB.

XRD was applied to analyze the mineral phases of hydration products in SCPB with 10%, 20%, and 30% of slag powder dosage, respectively, and the results are shown in Figure 6. Mica, chlorite, and quartz are the main natural mineral phases in the tailings; dicalcium silicate is the main mineral phase of slag powder; AFt is the mineral phase of hydration products. There are two main reactions evident in the cement–slag composite cementitious system: cement reaction and the slag reaction. In the SCPB slurry, the cement first reacts with water to produce hydration products such as C-S-H and Ca(OH)_2_ (CH). As the cement hydrates, the continuous generation of CH causes the pH of the slurry to gradually increase and starts to stimulate the activity of the slag powder. The OH^−^ in the alkaline environment then decomposes the glassy structure of the slag powder, which in turn produces hydration products such as C-S-H and AFt.

The mineral phases of C_3_S and C_3_A, which are highly reactive in cement, were not detected in any of the three sets of results shown in Figure 6, indicating that these two substances were completely hydrated to produce C-S-H gels and AFt crystals. The high diffraction peak of C_2_S indicates that some amount of this substance still existed in the SCPB. This is due to the low activity of C_2_S, which requires a higher alkaline environment for the hydration reaction to happen. Overall, the highest C_2_S diffraction peaks and the lowest AFt diffraction peaks were observed in the SCPB with 30% slag powder dosage. This was due to the relatively lower cement dosage caused by the high slag powder dosage, which in turn led to an alkaline environment, provided by cement hydration, that cannot fully react with C_2_S. The lowest diffraction peak of C_2_S and the highest diffraction peak of AFt were observed in the SCPB with 20% slag powder dosage. This is due to the disaggregation of the glassy structure of the slag powder, along with the release of AlO_4_^5−^ and Ca^2+^ by the CH generated by the hydration of the cement. These ions fully react with gypsum to form AFt, which decreases the C_2_S content and increases the AFt content in SCPB. The above analysis shows that the appropriate slag powder dosage can promote the hydration reaction of cement in the cement–slag composite cementitious system, while the cement hydration products in turn promote the decomposition of slag powder. This is the reason why the SCPB containing 20% slag powder had the highest UCS.

#### 3.2.2. FT-IR

**Figure 7:**
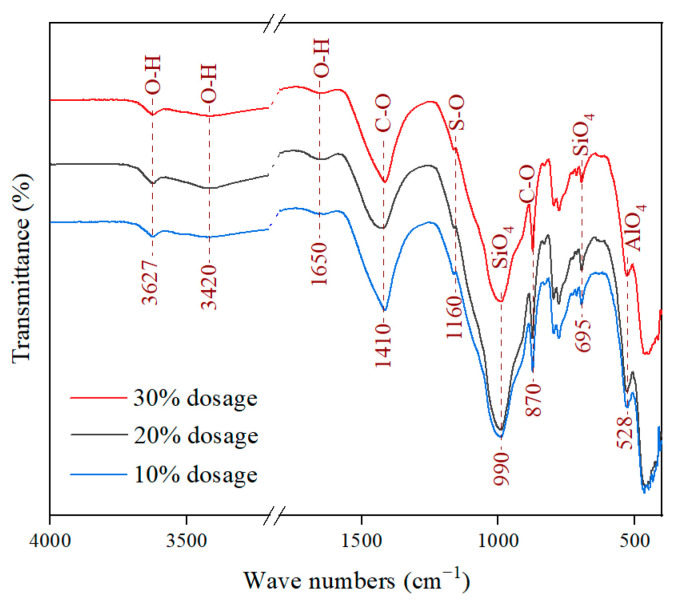
FT-IR results of SCPB.

FT-IR was used to analyze the molecular structures of hydration products in SCPB with slag powder dosages of 10%, 20%, and 30%, respectively, and the results are shown in Figure 7. The O-H bond represents to some extent the existence of water molecules and OH^−^ in the hydration products, and its characteristic peaks are mainly those of stretching vibrations in the range from 3000 cm^−1^ to 3750 cm^−1^ and the characteristic peaks of bending vibrations located near 1600 cm^−1^. The characteristic peaks of the stretching vibrations of the O-H bond in this study were located at 3627 cm^−1^ and 3420 cm^−1^, and the characteristic peaks of the bending vibrations were located at 1650 cm^−1^. From the characteristic peaks of the O-H bond, it can be seen that the infrared transmission rate of SCPB with 20% slag powder dosage was lower than that of the other two slag powder dosages of SCPB, indicating that more hydration products such as AFt and C-S-H were generated after the 20% slag powder dosage was added to the cement, which in turn reduced the transmission rate of the peak position. This result is in agreement with the XRD analysis. The characteristic peaks at 1410 cm^−1^ and 870 cm^−1^ are those of the stretching vibration and bending vibration of C-O bonds in CO_3_^2−^, mainly from dolomite and carbonation products in the raw material.

The characteristic peak at 1160 cm^−1^ is from the bending vibration of the S-O bond of SO_4_^2−^ in gypsum. The characteristic peak is extremely small, which indicates that the gypsum in the cement is almost completely involved in the hydration reaction. The characteristic peaks near 990 cm^−1^ and 695 cm^−1^ are the vibrational systolic peaks of silicon–oxygen tetrahedra (SiO_4_); the characteristic peaks near 528 cm^−1^ are the vibrational systolic peaks of aluminum–oxygen tetrahedra (AlO_4_). Both SiO_4_ and AlO_4_ are derived from the depolymerization of glassy structures in slag powder. The OH^−^ generated by the hydration of cement breaks the Si-O and Al-O bonds in the glassy structure of slag powder to form SiO_4_ and AlO_4_, and then the glassy structure is continuously depolymerized so that the SiO_4_ and AlO_4_ in the liquid phase gradually increase. These two substances further react with gypsum in cement to form hydration products such as C-S-H and C-A-S-H gels and AFt crystals. Overall, the SCPB with 20% slag powder dosage had the lowest transmittance of SiO_4_ and AlO_4_, which indicates that the depolymerization of slag powder in SCPB at this proportion was the most complete and generated the most hydration products. This is in agreement with the XRD results and further explains why the UCS of SCPB at this proportion was the highest. In the cement–slag composite cementitious system, when the cement and slag powder reach optimal proportions, it can promote the complete hydration of cement and fully activate the activity of slag powder, which gives the composite cementitious system optimal cementitious ability.

#### 3.2.3. TG-DTG

**Figure 8:**
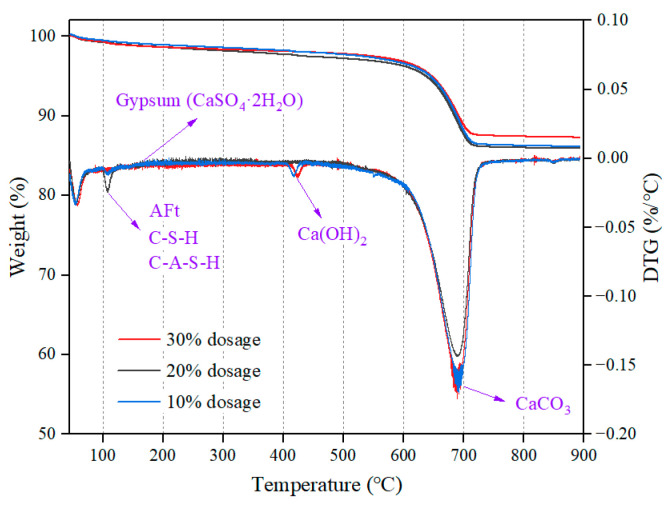
TG-DTA results of SCPB.

The results of TG-DTG analysis for SCPB with slag powder dosages of 10%, 20%, and 30% are shown in Figure 8. The mass loss below 500 °C was mainly from the dissipation of crystal water, interlayer water, and hydroxy groups from the structure of the hydration product. Among these, the mass loss below 240 °C resulted from the evaporation of crystal water and interlayer water; the mass loss in the range from 240 °C to 500 °C was mainly due to the dissipation of hydroxyl groups in the structure [43,44,45]. The mass loss in the range from 500 °C to 800 °C was mainly due to the dissipation of CO_2_ from the carbonate product.

The mass loss was more pronounced around 108 °C for the SCPB specimens. This loss peak corresponds to the evaporation of crystal water in C-S-H gels, C-A-S-H gels, and AFt crystals. Comparing the DTG curves of SCPB with different slag powder dosages, it was found that the SCPB with slag powder dosage of 20% had the largest loss peak, indicating that the SCPB with this slag powder dosage had the highest rate of mass loss and the largest mass loss at about 108 °C. This demonstrates that the SCPB with slag powder dosage of 20% had the greatest quantity of hydration products. A less pronounced loss peak was observed around 165 °C, which was due to mass loss from the decomposition and water loss of gypsum. This indicates the existence of a tiny amount of incompletely reacted gypsum in the SCPB. The mass loss around 420 °C was due to the decomposition of CH. The mass loss of SCPB with slag powder dosage of 10% or 30% was more significant, but there was almost no mass loss of SCPB with slag powder dosage of 20%, which indicates that the SCPB with slag powder dosage of 20% contained almost no CH. The slag powder can participate in the hydration reaction only after the disaggregation of the glass structure. The disaggregation of the glass structure requires the consumption of CH, and a lower content of CH indicates that the disaggregation of the glass structure of slag powder is more complete. The disaggregation of glass structure produces hydration products such as AFt and C-S-H, which is the main reason for the mass loss of SCPB with slag powder dosage of 20% at about 108 °C. These results corroborate those of the XRD and FT-IR analysis.

### 3.3. Microstructure

**Figure 9:**
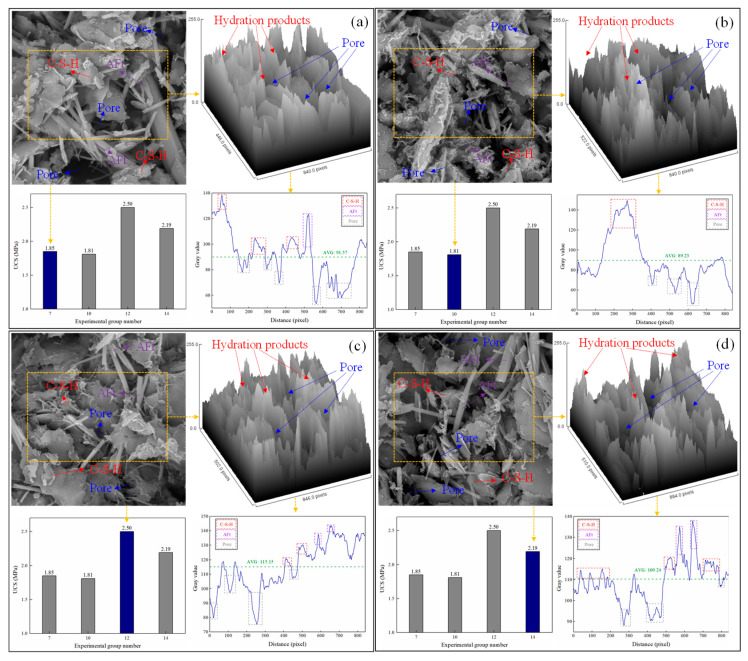
Microstructure and grayscale analysis of SCPB: (**a**) group 7, (**b**) group 10, (**c**) group 12, (**d**) group 14.

The microstructure images of SCPB were obtained using SEM. The grayscale and UCS analysis of different microstructures are shown in Figure 9. The hydration reaction produced a large number of AFt crystals and CSH gels. The AFt crystals appear as needles and rods in the pore structure and are well developed. Most of the Aft crystals have a diameter of more than 0.3 nm and some of them have a diameter of 1 nm, while a few newly developed crystals are smaller in diameter. CSH gels are amorphously distributed in SCPB and develop and accumulate in solid particles and AFt crystals, etc. AFt crystals are interwoven in the pore structure and form a network structure. The CSH gel is attached to the network structure, which enhances the strength of the structure. A network structure consisting of AFt crystals and CSH gels fills between the solid particles and binds them together, which both reduces the porosity and enhances the strength of the SCPB.

Comparing Figure 9a–c, it can be seen that the average gray value of the microstructure of SCPB increased from 89.23 and 91.57 to 115.15 and the strength of SCPB increased from 1.81 MPa and 1.85 MPa to 2.50 MPa when the slag powder dosage was changed from 10% and 30% to 20%. This represents an increase in the average gray value of 29.0% and 25.8% and an enhancement in the UCS of 38.1% and 35.1%, respectively. Also observing the microstructure image of SCPB, it can be seen that when the average gray value is low, the structure of SCPB is loose and there are many pores inside. This weakens its macroscopic strength. When the average gray value is higher, the structure of SCPB is more compact with fewer internal pores, and the macroscopic strength is higher. Comparing Figure 9c,d, it can be seen that the average gray value of SCPB increased from 109 to 115 and the UCS of SCPB increased from 2.19 to 2.50 MPa when the slurry mass fraction is increased from 68% to 71% and the underflow productivity decreased from 75% to 65%. Furthermore, the corresponding microstructure images indicate that increasing the slurry mass fraction and decreasing the underflow productivity reduces the pores inside the SCPB, which makes the structure of SCPB more compact. This is the reason for the enhancement of the UCS of SCPB.

## 4. Prediction and Optimization Based on Machine Learning

### 4.1. Comparison and Selection of Networks

#### 4.1.1. Long Short-Term Memory Neural Network

**Figure 10:**
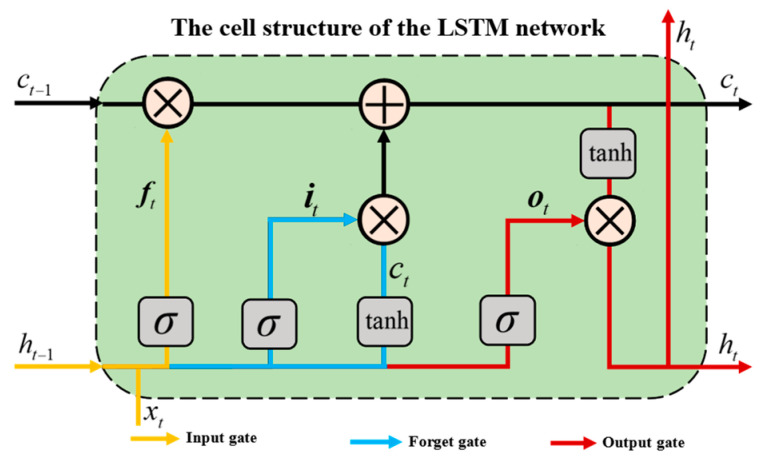
The cell structure of the LSTM network.

A long short-term memory neural network (LSTM) is a commonly used deep learning algorithm for modeling sequential data, which can solve the problem of vanishing gradients encountered in conventional RNN algorithms. Typically, LSTM consists of input gates, forget gates, and output gates, each composed of a sigmoid neural network and an element-wise multiplication operation. In addition, LSTM uses a structure called “cell state” to store and transmit information. The cell state can be regarded as the “memory” of LSTM, as it can retain previous information and pass it on to subsequent time steps. During the training phase, LSTM updates the parameters in the model using the backpropagation algorithm to minimize the error between predicted values and actual values. During the prediction phase, LSTM takes the current input and the state of the previous time step as inputs, and outputs the predicted value for the current time step [46,47,48]. The architecture of the LSTM network is depicted in Figure 10.

#### 4.1.2. Performance Metrics

Different model parameters lead to different prediction effects for models, and the prediction effects of different models are also different. To evaluate the prediction effects of the different models, the following common performance metrics were applied to compare their prediction performance:

(2) $$\mathrm{RMSE}=\sqrt{\frac{1}{n}\sum_{i=1}^{n}{\left[y_{i}-f\left(x_{i}\right)\right]}^{2}}$$

(3) $$R=\frac{\sum_{i=1}^{n}[\left(y_{i}-\bar{y_{i}}\right)\left(f\left(x_{i}\right)-\bar{f\left(x_{i}\right)}\right]}{\sqrt{\sum_{i=1}^{n}{\left(y_{i}-\bar{y}\right)}^{2}}\cdot \sqrt{{\sum_{i=1}^{n}\left(f\left(x_{i}\right)-\bar{f(x_{i}}\right))}^{2}}}$$

(4) $$\mathrm{VAF}=\left[1-\frac{\mathrm{VAR}\left(y_{i}-f\left(x_{i}\right)\right)}{\mathrm{VAR}\left(y_{i}\right)}\right]\times 100$$

#### 4.1.3. Adjustment of Parameters in Different Neural Network

In this research, a back propagation neural network (BPNN), extreme learning machine (ELM), and radial basis function neural network (RBFNN) were used for comparison with LSTM to determine the optimal model that can map the relationship between the four variables and UCS. Due to limitations of space, the formulas and derivation process of the above three neural networks are not discussed in this section; interested readers can refer to the references [49,50,51].

To ensure the fairness of the comparison and to avoid overfitting, above all, neural networks are networks with a single hidden layer. The initial learning rate, target error, and max iterations were set to 0.005, 0.001, and 100, according to references [24,25,31], with a large number of pre-tuning tests. Moreover, the numbers of input layer nodes and output layer nodes correspond to the numbers of input variables and output variables, respectively. In research using neural networks for prediction, the number of hidden layer neurons affects the computational speed of the neural network and the accuracy of the prediction results. If the number of hidden layer nodes is too few, the neural network may not be trained or poor performance may result; if the number of hidden layer nodes is too many, although the prediction error of the neural network can be reduced, it can extend the network training time [46,52,53,54]. Too many hidden layer nodes can make the neural network very easily fall into local optimum during the training process, which is also one of the reasons for overfitting. In addition, the spread factor is also an important parameter in RBFNNs, referring to the expansion factor of the radial basis function. Theoretically, the larger the value of the spread factor, the smoother will be the output of the RBFNN [50,55,56]. However, a large spread factor can lead to huge computational effort and overfitting [57,58,59]. To search for the optimal number of hidden layer nodes and spread factor for the RBFNN, this research used Equation (2) to find the optimal number of hidden layer nodes, and a trial-and-error method to identify the optimal spread factor in the interval (1,16) according to previous experience of parameter adjustment.

(5) $$\mathrm{Number}\;\mathrm{of}\;\mathrm{hidden}\;\mathrm{layer}\;\mathrm{nodes}\;=\;\sqrt{m+n}+l$$

where *m* is the number of inputs, *n* is the number of outputs, and *l* is the adjustable constant on the interval (1,10).

**Figure 11:**
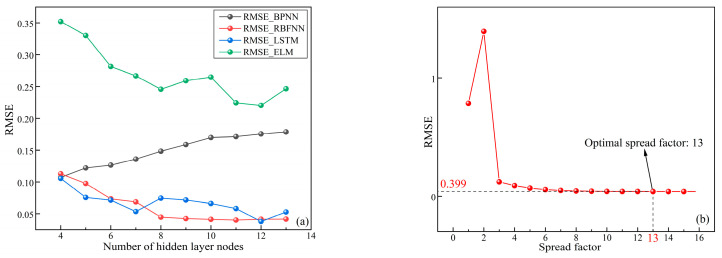
The optimization search results of the parameters: (**a**) hidden layer nodes, (**b**) spread factor.

The 51 UCS samples obtained from the experiments in Section 2 were randomly selected as the training set according to the principle of training set: validation set = 8:2 (quantity ratio is 40:11), to find the optimal number of hidden layer nodes and spread factor. The optimization search results of the parameters are shown in Figure 11. As shown in Figure 11a, the RMSE of ELM and BPNN for the training set was significantly higher than that of RBFNN and LSTM. The RMSE of ELM decreased with the increase in the number of hidden layer nodes when the RMSE was higher than 0.2; the RMSE of BP increased with the increase of the number of hidden layer nodes when the RMSE was higher than 0.1; the RMSE of RBFNN decreased with the increase of the number of hidden layer nodes when the RMSE was lower than 0.1; the RMSE of LSTM showed a fluctuating decreasing trend with the increase of the number of hidden layer nodes when the RMSE of LSTM was close to that of RBFNN. Furthermore, it can be seen that the RMSEs of BPNN, RBFNN, ELM and LSTM attained minimum values of 0.1071, 0.0401, 0.0378, and 0.2203 when the numbers of hidden layer nodes were 4, 11, 12, and 12, respectively. Figure 11b shows that during the training of RBFNN, the RMSE increased when the spread factor changed from 1 to 2, then decreased rapidly. When the spread factor reached 13, the RMSE of RBFNN for the training set attained the minimum value of 0.399. The structures and parameters of the four neural networks are summarized in Table 4.

#### 4.1.4. Comparison of Prediction Results of Different Neural Networks

**Figure 12:**
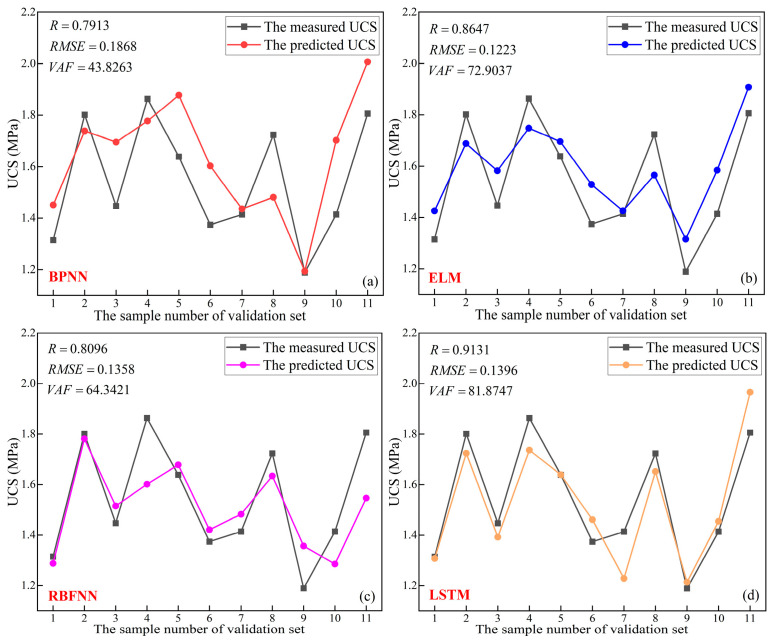
Prediction results of neural networks: (**a**) BPNN, (**b**) ELM, (**c**) RBFNN, (**d**) LSTM.

The 11 sets of samples from the validation set were substituted into the neural network already trained in Section 4.1.3 for prediction of UCS, and the results are shown in Figure 12. As shown in Figure 12a, the R, RMSE, and VAF of the prediction results of BPNN for the validation set were 0.7913, 0.1868, and 43.8263, respectively. Figure 12b reports that the R, RMSE, and VAF of the prediction results of ELM for the validation set were 0.8647, 0.1223 and 72.9037, respectively. According to Figure 12c, the R, RMSE, and VAF of the prediction results of RBFNN for the validation set were 0.8096, 0.1358, and 64.3421, respectively. From Figure 12d, it can be seen that the R, RMSE, and VAF of the prediction results of LSTM for the validation set were 0.9131, 0.1396, and 81.8747, respectively.

Performance metrics of different models are shown in Table 5 [23]. Although ELM and LSTM have the same total rank, the R and VAF of LSTM are both better than for ELM. This indicates that the predicted UCS of LSTM has a better fit with the measured UCS.

### 4.2. Optimization of LSTM

**Figure 13:**
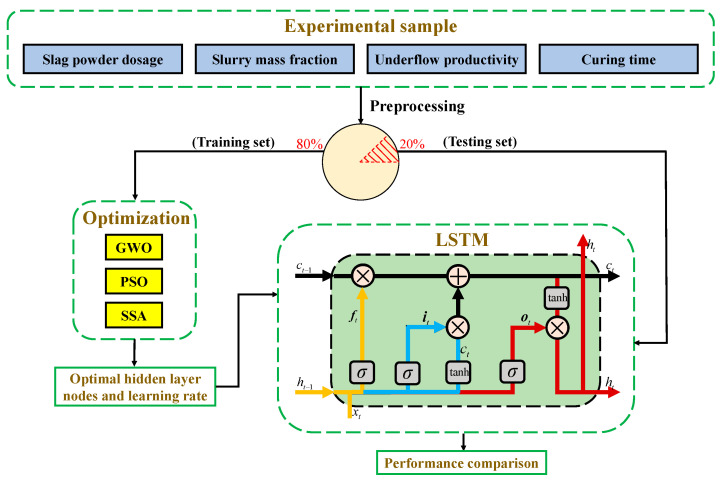
The optimization and modeling process.

Previous studies have shown that the number of hidden layer nodes and the learning rate have a significant effect on the performance of LSTM [46,60,61,62]. The learning rate determines whether and when a neural network can converge. In other words, an appropriate learning rate enables the neural network to converge to the target value at the right time. If the learning rate is too low, the loss function changes too slowly, which increases the convergence complexity of the network significantly and makes it easy to trap the network at the local optimum. If the learning rate is too high, the loss function crosses the global optimum directly while the gradient oscillates back and forth around the optimum and may not even converge. The effect of the number of hidden layer nodes on the LSTM is discussed in Section 4.1.3 and is not repeated here. To improve the performance of LSTM, the hidden layer nodes and the learning rate are optimized using GWO, PSO, and the sparrow search algorithm (SSA), which have been widely used in industry and academia with excellent results [63,64,65]. Due to the limitations of space, the principles and formulas of the optimization algorithm are not repeated in this section, and interested readers can refer to the references [66,67,68,69]. The optimization and modeling process is shown in Figure 13.

#### 4.2.1. Results of GWO-LSTM

**Figure 14:**
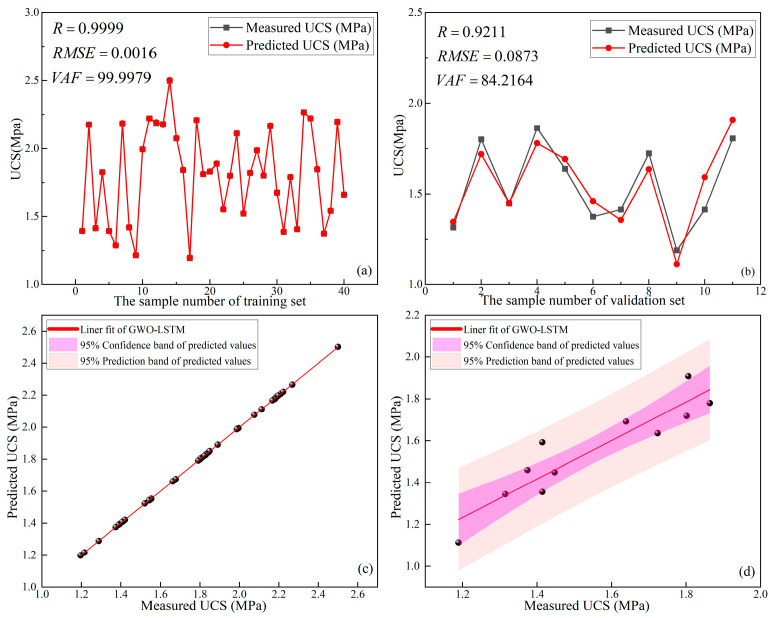
Prediction results of GWO-LSTM: (**a**) training set, (**b**) validation set, (**c**) linear fit of training set, (**d**) linear fit of validation set.

The optimal parameters were obtained after using LSTM improved by GWO (GWO-LSTM): the learning rate was 0.0162 and the number of hidden layer nodes was 26. The prediction results of GWO-LSTM for the UCS of the training set and validation set are shown in Figure 14. From Figure 14a, it can be seen that the prediction accuracy of the GWO-LSTM in the training set was extremely high (R = 0.9999, RMSE = 0.0016, and VAF = 99.9979). From Figure 14b, it can be seen that the prediction accuracy of GWO-LSTM for the validation set was relatively poor (R = 0.9211, RMSE = 0.0873, and VAF = 84.2164) but still higher than that of the unoptimized LSTM described in Section 4.1.4. Figure 14c,d shows the linear fit of the GWO-LSTM to the predicted and measured UCS of the training and validation sets, respectively. Figure 14c,d shows that the width of the 95% confidence interval and the width of the 95% prediction interval for the validation set are much narrower than those of the training set, indicating that the fit of the data in the validation set under GWO-LSTM was much less than the training set. This means that the GWO-LSTM obtained excellent training results, but the difference between the prediction results of the validation set and the training set was relatively large. This phenomenon may have been caused by the overfitting of the GWO-LSTM in the calculation.

#### 4.2.2. Results of PSO-LSTM

**Figure 15:**
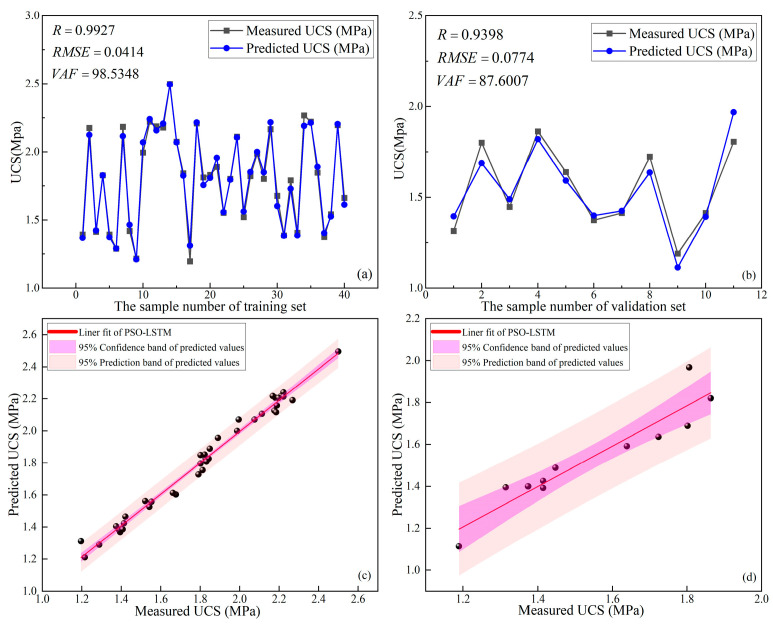
Prediction results of PSO-LSTM: (**a**) training set, (**b**) validation set, (**c**) linear fit of training set, (**d**) linear fit of validation set.

The optimal parameters were obtained after using LSTM improved by PSO (PSO-LSTM): the learning rate was 0.0381 and the number of hidden layer nodes was 37. The prediction results of PSO-LSTM for the UCS of the training set and validation set are shown in Figure 15. From Figure 15a, it can be seen that the training performance of the PSO-LSTM in the training set was good (R = 0.9927, RMSE = 0.0414, and VAF = 98.5348). From Figure 15b, it can be seen that the prediction accuracy of PSO-LSTM for the validation set was relatively poor (R = 0.9398, RMSE = 0.0774, and VAF = 87.6007) but still higher than that of the unoptimized LSTM described in Section 4.1.4. Figure 15c,d shows the linear fits of the PSO-LSTM to the predicted UCS and the measured UCS of the training and validation sets, respectively. As shown in Figure 15c,d, although the width of the 95% confidence interval and the width of the 95% prediction interval of the validation set are narrower than those of the training set, the distribution of sample points in the validation set is basically concentrated in the vicinity of the fit line. This indicates that the generalization of PSO-LSTM is average, but better than that of GWO-LSTM. Moreover, the gap between the confidence interval and the prediction interval of the training sets and validation sets was significantly lower than that for GWO-LSTM. In conclusion, although the generalization ability of PSO-LSTM has some room for improvement, it can effectively train the dataset and make predictions for UCS, with some performance improvements compared with GWO-LSTM.

#### 4.2.3. Results of SSA-LSTM

**Figure 16:**
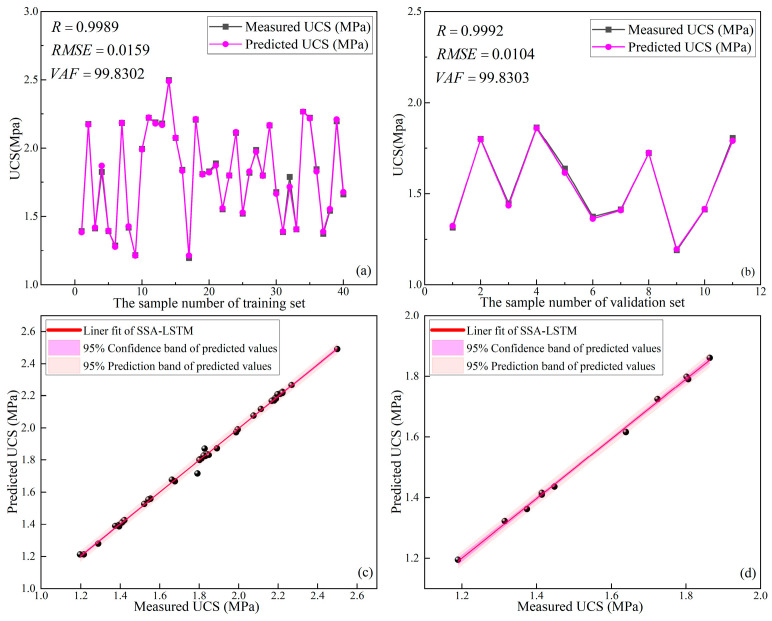
Prediction results of SSA-LSTM: (**a**) training set, (**b**) validation set, (**c**) linear fit of training set, (**d**) linear fit of validation set.

The optimal parameters were obtained after using LSTM improved by SSA (SSA-LSTM): the learning rate was 0.0146 and the number of hidden layer nodes was 19. The prediction results of SSA-LSTM for the UCS of the training set and validation set are shown in Figure 16. From Figure 16a, it can be seen that the training performance of the SSA-LSTM in the training set was good (R = 0.9989, RMSE = 0.0159, and VAF = 99.7831). From Figure 16b, it can be seen that the prediction accuracy of SSA-LSTM for the validation set was relatively poor (R = 0.9992, RMSE = 0.0144 and VAF = 99.8303) but still higher than that of the unoptimized LSTM described in Section 4.1.4. Figure 16c,d shows the linear fits of the SSA-LSTM to the predicted UCS and the measured UCS of the training and validation sets, respectively. As shown in Figure 16c,d, the 95% confidence intervals and 95% prediction intervals of the training sets and validation sets are basically the same, which indicates that SSA-LSTM is not only able to characterize accurately the complex mapping relationship between the experimental variables and UCS, but also has excellent generalization ability and strong robustness. A comprehensive analysis of Figure 16 shows that the SSA-LSTM has excellent prediction performance and it is the optimal model among the three models used for comparison.

#### 4.2.4. Analysis and Discussion of Results

**Figure 17:**
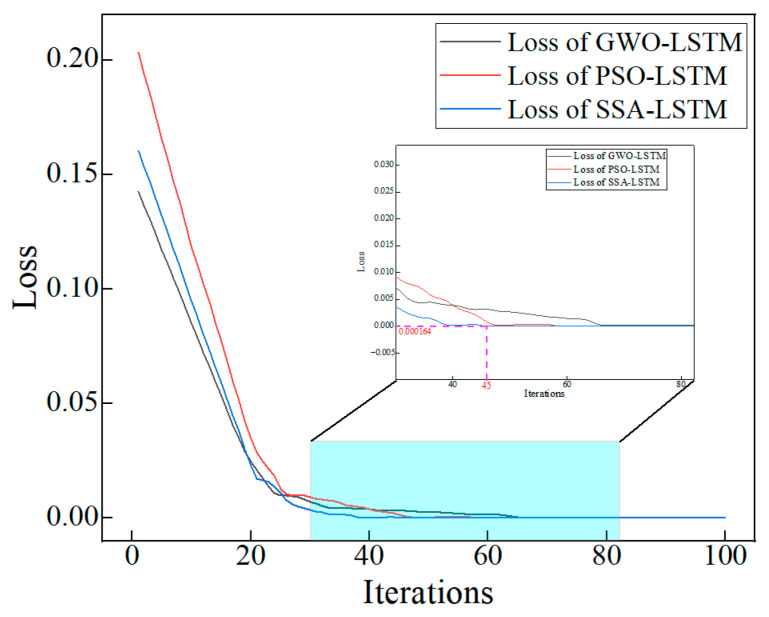
The training process of the optimized LSTMs.

The training process of the above three optimized LSTMs was tracked by using the half mean square error as the loss function, as shown in Figure 17. From Figure 17, it can be seen that when the iterations reach 45 the loss of SSA-LSTM is the first to reach a steady state and reach a minimum of 0.000164. This shows that SSA-LSTM can minimize the error between the measured UCS and predicted UCS initially during the training process. The loss by GWO-LSTM and PSO-LSTM was also minimized, but significantly lagged behind SSA-LSTM. The above results show that SSA-LSTM has excellent prediction performance compared with GWO-LSTM and PSO-LSTM.

**Figure 18:**
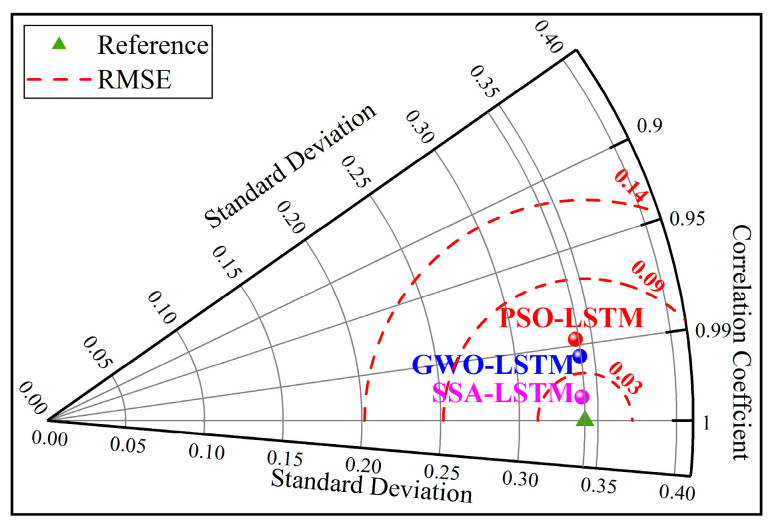
Taylor diagram of different models.

The predictive performance of the different models can be seen in Figure 18 [70,71]. The SSA-LSTM had not only the lowest RMSE but also the highest R (the standard deviations of the three models are almost the same), which indicates that SSA-LSTM has the optimal predictive performance. In summary, SSA-LSTM has excellent prediction accuracy, generalization, and robustness in the prediction of UCS of SCPB, and can be used as a new intelligent tool in filling mining.

## 5. Conclusions

To improve the utilization of ultrafine tailings and optimize the mechanical properties of SCPB, a series of experiments and tests were conducted to analyze the effects of different factors on the strength of SCPB. Furthermore, prediction of SCPB strength under multiple factors was carried out by combining machine learning. The main conclusions are as follows:

(1) The strength of SCPB exhibited an initial increase followed by a decrease with an increase in slag powder dosage. It showed an increase with the rise in slurry mass fraction and a decrease with the increase in underflow productivity. The combined influence of slag powder dosage and slurry mass fraction had the most pronounced effect on UCS, whereas the coupling effect of slurry mass fraction and underflow productivity had the least impact on UCS.
(2) The generation of hydration products of SCPB with slag powder dosage of 20% was the highest, for the following reasons. The CH generated by the hydration of cement caused complete depolymerization of the glass bodies of slag powder, and released AlO_4_^5−^ and Ca^2+^. These ions fully reacted with gypsum in the cement to produce a large quantity of hydration products, and the consumption of CH by slag powder further promotes the hydration of the cement.
(3) The LSTM model developed in this study demonstrates the highest prediction accuracy for the strength of SCPB under multi-factor conditions. The model achieved R, RMSE, and VAF values of 0.9131, 0.1396, and 81.8747, respectively. Through optimization using the SSA algorithm, the LSTM model’s performance was further enhanced, resulting in an improvement of 9.4% in R, 88.6% in RMSE, and 21.9% in VAF.

## Figures and Tables

**Table 1:** Chemical compositions of cementitious materials.

Chemical Composition	SiO_2_	CaO	MgO	Fe_2_O_3_	Al_2_O_3_	SO_3_	TiO_2_
Tailings	67.1%	2.51%	0.65%	2.17%	16.73%	0.9%	0.25%
Cement	20.35%	62.20%	4.22%	3.17%	4.34%	2.54%	/
Slag powder	27.51%	43.24%	8.09%	0.38%	16.25%	1.51%	0.32%

**Table 2:** RSM experimental design.

Factors	Horizon Codes		
	−1	0	1
Slag powder dosage (*X*_1_)	10	20	30
Slurry mass fraction (*X*_2_)	65	68	71
Underflow productivity (*X*_3_)	75	65	55

**Table 3:** The experimental results.

Number	Factors/%			Measured UCS/MPa		
	*X*_1_	*X*_2_	*X*_3_	*Y*_1_	*Y*_2_	*Y*_3_
1	20	68	65	1.39	1.79	2.17
2	20	71	75	1.45	1.89	2.27
3	10	68	75	1.31	1.68	2.00
4	20	68	65	1.41	1.80	2.18
5	10	68	55	1.37	1.84	2.20
6	20	68	65	1.41	1.80	2.18
7	30	65	65	1.22	1.55	1.85
8	20	68	65	1.39	1.81	2.18
9	30	71	65	1.41	1.82	2.21
10	10	65	65	1.19	1.52	1.81
11	10	71	65	1.42	1.83	2.22
12	20	71	55	1.64	2.11	2.50
13	30	68	55	1.39	1.86	2.22
14	20	68	65	1.41	1.80	2.19
15	20	65	55	1.37	1.72	2.07
16	20	65	75	1.20	1.54	1.83
17	30	68	75	1.29	1.66	1.99

**Table 5:** The performance indices of different networks.

Network	Results			Rank Values			Total Rank
	R	RMSE	VAF	R	RMSE	VAF	
BPNN	0.7913	0.1868	43.8263	1	1	1	3
ELM	0.8647	0.1223	72.9037	3	4	3	10
RBFNN	0.8096	0.1358	64.3421	2	3	2	7
LSTM	0.9131	0.1396	81.8747	4	2	4	10

**Table 4:** The structures and parameters of four neural networks.

Models	Initial Learning Rate	Target Error	Max Iterations	Number of Hidden Layers	Structure	Spread Factor
LSTM	0.005	0.001	100	1	4-12-1	/
BPNN					4-4-1	/
ELM					4-12-1	/
RBFNN					4-13-1	13

## Data Availability

The data generated in this study are available upon request.
